# An underestimated pathogen: *Corynebacterium* species

**DOI:** 10.1128/jcm.01552-24

**Published:** 2025-08-20

**Authors:** Brooks I. Mitchell, John E. Markantonis

**Affiliations:** 1 Department of Pathology, Northwestern University Feinberg School of Medicine 12244, Chicago, Illinois, USA; 2 Clinical Microbiology Laboratory, Northwestern Memorial Hospital 24560, Chicago, Illinois, USA; 3 Department of Pathology & Laboratory Medicine, Brody School of Medicine, East Carolina University 12278, Greenville, North Carolina, USA; Vanderbilt University Medical Center, Nashville, Tennessee, USA

**Keywords:** *Corynebacterium*, coryneform gram-positive rods, diphtheria

## Abstract

*Corynebacterium* species are a diverse group of organisms historically considered to be non-pathogenic, outside of the *C. diphtheriae* complex. Over the last few decades, this belief has been disproven with many notable non-diphtheriae *Corynebacterium* species being found to be pathogenic, often in certain clinical scenarios and/or anatomical sites. *C. striatum* and *C. jeikeium* are responsible for a large portion of bloodstream infections and orthopedic infections related to coryneform Gram-positive rods (GPRs). Eye and ear infections have commonly been attributed to *C. macginleyi* and *C. otitidis*, respectively. Pneumonia in critically ill and immunosuppressed individuals has been frequently reported by the *C. propinquum*/*pseudodiphtheriticum* group and occasionally in *C. striatum. C. urealyticum* is the primary pathogen associated with encrusted cystitis. Granulomatous lobular mastitis and breast abscesses have a strong association with *C. kroppenstedtii*. Erythrasma (*C. aurimucosum*/*minutissimum* group), trichobacteriosis (*C. flavescens*), and hidradenitis suppurativa are cutaneous disorders caused by or associated with *Corynebacterium* species. Biofilm formation by these bacteria leads to hardware/medical device-associated infections involving endovascular catheters, cerebrospinal fluid shunts, peritoneal dialysis catheters, and prosthetic joints. Clinical microbiology laboratories must be aware of this and optimize laboratory identification and reporting of these organisms when appropriate. Matrix-associated laser/adsorption ionization time-of-flight mass spectrometry, currently available in most large clinical microbiology laboratories, offers laboratories the ability to rapidly, accurately, and affordably accomplish this task.

## INTRODUCTION

The genus *Corynebacterium* encompasses Gram-positive organisms with much heterogeneity in cellular and colony morphology, growth requirements, and environmental predilections observed among its numerous species. Organisms of this genus are described to be mostly catalase positive, facultatively anaerobic, and non-motile. Structurally, the cellular envelope of *Corynebacterium* spp. is complex, with esterified short-chain alpha-branched and beta-hydroxy fatty acids (corynomycolates) covalently bound to the peptidoglycan wall, ultimately forming a mycolyl-peptidoglycan complex (1). The presence of these complexes contributes to stress resistance and pathogenicity; functional similarities are seen in their close relatives, *Mycobacterium* spp. (2). With over 160 species identified (https://lpsn.dsmz.de/genus/corynebacterium accessed May 4, 2025), the diverse genus of *Corynebacterium* includes organisms with known relevance in the fields of human and veterinary medicine, as well as biotechnology (3). Historically, *C. diphtheriae, C. ulcerans, *and* C. pseudotuberculosis* were the pathogenic species characterized as being diphtheria toxin (DT)-producing. However, later, the “*C. diphtheriae* complex” was defined and included other potentially DT-producing species: *C. belfantii*, *C. rouxii*, and *C. silvaticum* (4). These notable pathogens have a significant impact on public health worldwide, with their host range including humans (*C. diphtheriae, C. belfantii,* and *C. rouxii*), as well as wild and domesticated mammals (*C. ulcerans*, *C. pseudotuberculosis*, and *C. silvaticum*) (4). With the improvement of identification and molecular phylogenetic modalities, the understanding of ecological/host niches and pathogenicity factors of otherwise “commensal” *Corynebacterium* species has advanced, particularly the understanding of species isolated from human samples, which has resulted in a growing concern about their clinical significance. In this review, we highlight recent literature that underscores these concerns.

## PATHOGENICITY

### Bloodstream infections

As common commensal skin flora, *Corynebacterium* spp. were once almost universally disregarded as contaminants when isolated in blood culture. This is often the case; however, it is now known that true cases of bloodstream infections due to *Corynebacterium* spp. do happen (5). When this occurs, the resulting morbidity and mortality rates are high with *C. jeikeium* and *C. striatum,* the two species most commonly associated with bacteremia (5). This is most commonly seen in patients with neutropenia and/or malignancy, especially in hematological cancers (5). Both of these species are known to produce biofilm, which can lead to catheter-associated bacteremia (5, 6). Bloodstream infections have been reported due to *C. accolens*, *C. afermentans* subsp. *afermentans, C. afermentans* subsp. *lipophilum, C. amycolatum, C. argentoratense, C. aurimucosum*/*minutissimum* group*, C. coyleae, C. diphtheriae complex*, *C. falsenii, C. freneyi, C. glucuronolyticum, C. imitans, C. kroppenstedtii, C. mucifaciens, C. propinquum/pseudodiphtheriticum* group*, C. resistens*, *C. timonense*, *C. tuscaniae*, *C. urealyticum, C. ureicelerivorans, C. xerosis,* and several others (7–10). Infective endocarditis of both native and prosthetic heart valves has been reported due to *Corynebacterium* spp., the majority are due to *C. jeikeium* and *C. striatum* (8). Cases of infective endocarditis have also been attributed to other species in this genus, including *C. amycolatum*, *C. aurimucosum*/*minutissimum* group*, C. coyleae, C. diphtheria complex, C. macginleyi, C. propinquum*/*pseudodiphtheriticum* group*, C. simulans, C. timonense, C. urealyticum, *and* C. xerosis *(7–9).

### Central nervous system infections

Infections of the central nervous system (CNS) are rare but do occur. Most cases are postoperative in nature, but rare cases of spontaneous infection have been reported (7, 11). Cases have been attributed to *C. afermentans* subsp. *lipophilum, C. aurimucosum*/*minutissimum* group, *C. jeikeium, C. propinquum*/*pseudodiphtheriticum *group*, C. resistens, *and *C. xerosis *(7). *C. jeikeium, C. striatum,* and *C. xerosis* have been implicated in infections of cerebrospinal fluid (CSF) shunts on multiple occasions (11).

### Cutaneous and soft tissue infections

#### Cellulitis and wound infections

Colonization of skin and mucosal surfaces by *Corynebacterium* spp. is common. When infection occurs, it is generally opportunistic, occurring in immunosuppressed hosts where skin breakdown and/or trauma has occurred. *C. jeikeium, C. aurimucosum*/*minutissimum* group*, C. propinquum*/*pseudodiphtheriticum* group*, C. resistens*, and *C. striatum* are the most common causative agents (7)*. C. canis* and *C. freiburgense* have been recovered from skin wounds in immunocompetent individuals following dog bites (7). *C. kutscheri* was cultured from a wound following a rat bite (7). Skin ulcers have been reported due to *C. mycetoides* (7).

#### Cutaneous diphtheria

*C. diphtheriae* complex*, C. ulcerans, *and* C. pseudotuberculosis* are known to harbor the diphtheria toxin gene (*tox*) acquired by corynebacteriophage and are capable of causing diphtheria in humans (4). *C. diphtheriae* is spread human-to-human by droplet or direct contact, while *C. ulcerans* and *C. pseudotuberculosis* are zoonotic (4). Three recently described species have been identified within the *C. diphtheriae* species complex, which have the potential to cause diphtheria due to the ability to harbor the *tox* gene: *C. belfantii, C. rouxii*, and *C. silvaticum* (4). When these diphtheria toxin (DT)-producing *Corynebacterium* spp. have cutaneous involvement, cellulitis and/or ulcers may result (4). In humans, this can be seen with infections by *C. diphtheriae* complex and *C. ulcerans* (4). A thick, grayish pseudomembrane on the surface of the ulcer may be present when DT production occurs (4). Systemic complications of diphtheria toxin, such as myocarditis and nerve injury, are possible from both cutaneous and respiratory diphtheria (4). Non-DT-producing strains of these species can also result in non-healing cutaneous ulcers, which are more common than DT-producing strains in the present day due to vaccination (4). Vaccination is protective against DT-producing strains but is not effective against nontoxigenic strains of these species (4, 12). The ulcers from nontoxigenic strains of these species commonly have a fibrinous base with an erythematous, edematous, non-indurated, and unraised border (12). Prompt systemic antimicrobial treatment is necessary to avoid systemic and invasive complications in both toxigenic and nontoxigenic strains of these species (12).

#### Erythrasma

*Corynebacterium minutissimum* has been classically associated with a chronic superficial erythematous rash called erythrasma, which involves warm, moist intertriginous body spaces (13, 14). *C. minutissimum* can be misidentified for the closely related *C. aurimucosum*, this is especially true when using conventional phenotypic methods (15). This does cast some concerns about the reliability of the identification of *C. minutissimum* in historic cases where the bacteria were isolated in culture from clinical cases. The use of the *C*. *amycolatum*/*minutissimum* group when discussing these previous cases is likely the most accurate identification. *C. minutissimum* does produce coproporphyrin III, which fluoresces with a coral-pink coloration under UV light (13). Wood’s lamp illumination of skin lesions containing the bacteria or colonies grown on agar plates will reveal coral-pink fluorescence (13). The diagnosis of erythrasma is generally obtained by clinical presentation and physical examination findings, with culture-confirmed cases by *C. minutissimum* being sparse at best (13). When a microbiological culture of erythrasma skin lesions has been performed, other potential pathogens such as yeast and dermatophytes have been recovered (13). This sheds doubt on this cutaneous disease as a purely monomicrobial process. Treatment for erythrasma generally involves topical antimicrobial therapy; however, in severe cases, systemic antibiotics may be considered (13).

#### Granulomatous lobular mastitis

Granulomatous lobular mastitis is an uncommon disorder of the breast that typically presents in childbearing women as a unilateral breast mass with overlying skin changes (16, 17). It is often confused with malignancy given these clinical findings, but it is a benign inflammatory condition (16, 17). Recurrence is a hallmark of this disorder, which can make treatment challenging (16, 17). Treatment with antimicrobial courses, steroid therapy, and/or surgical excision is often required, especially in large lesions with relapse (16). Granulomatous lobular mastitis (GLM) is characterized by the presence of non-necrotizing granulomas within and surrounding lobules on histopathology of biopsied or excised breast tissue (17). An associated neutrophilic response with or without microabscesses can also be seen (17). In some of these cases, cystic spaces lined with neutrophils will be visible within a background of pyogranulomatous inflammation; this has been termed a cystic neutrophilic granulomatous mastitis (CNGM) pattern (17). Gram-positive rods (GPRs) consistent with *Corynebacterium* species have been identified within these cystic spaces (17). Microbiological cultures and sequencing studies often recover *Corynebacterium* species in GLM, especially in cases where a CNGM pattern has been seen on histopathology (16, 17). *C. kroppenstedtii* and the newly discovered *C. kroppenstedtii*-like isolates (*C. parakroppenstedtii* and *C. pseudokroppenstedtii*) are the most recovered organisms from GLM and likely have a role in the pathogenicity of this condition (18). Occasionally, other *Corynebacterium* spp. such as C. *amycolatum* and *C. tuberculostearicum* complex are recovered from cultures in cases of GLM (18).

#### Hidradenitis supportiva

Hidradenitis suppurativa (HS) is a chronic, inflammatory skin condition that results in recurring skin abscesses and sinus tracts in intertriginous body spaces (19). Currently, diagnosis is largely clinical in nature (19). Microbiological culture has historically been of low clinical utility in this disorder, as they are often reported as mixed skin or cutaneous flora (19). However, new research has shown that skin flora such as *Staphylococcus* spp. and *Corynebacterium* spp. may contribute to the condition (20). Systemic antimicrobial combination therapy with agents such as rifampin, clindamycin, moxifloxacin, and metronidazole appears to be effective in the treatment of HS (19).

#### Skin and soft tissue abscess

*C. amycolatum, C. diphtheriae complex, C. pyruviciproducens, C. striatum, *and* C. ulcerans* have been known to cause skin and soft tissue abscesses (7). In addition to GLM, *C. kroppenstedtii* can cause breast abscesses (7). These are often recurring and can be challenging to treat (16).

#### Trichobacteriosis

This infection was previously termed trichomycosis when the causative agent was thought to be fungal (21). With the recent knowledge that its etiology is likely bacterial, trichobacteriosis is now the preferred name for this dermatological condition (21). The majority of cases are associated with *Corynebacterium* spp. (21). It is a benign infection of hair follicles (21). It most often involves axillary hair; however, pubic and scalp hair may also be involved (21). Bacterial concretions adhering to hair shafts may lead to thickening of the hair or a change in hair coloration, most commonly to yellow, but red and black coloration may be seen (21). Under UV fluorescence, the hair follicles may have a neon yellowish-green coloration if caused by *C. flavescens* (21). Similar to erythrasma, the use of a Wood’s lamp may aid in diagnosis (21). Treatment with topical antibiotics or shaving the affected hair is generally effective (21).

### Eye and ear infections

#### Conjunctivitis, keratitis, and corneal ulcers

The normal microbiome of the ocular surface often contains *Corynebacterium* species (22)*. C. macginleyi* is a common inhabitant of the ocular surface and is often the predominant species found there (22). It is also the most common cause of conjunctivitis and keratitis by a *Corynebacterium* species (22). *C. accolens, C. amycolatum, C. bovis, C. jeikeium, C. mastitidis*, *C. propinquum*/*pseudodiphtheriticum* group, *C. striatum*, and *C. xerosis* have been associated with infections of the eye (22). Ocular diphtheria is rare in Western countries with high vaccination rates, but when it does occur, it can result in corneal perforation (23).

#### Otitis media and otitis externa

*Staphylococcus aureus* and *Pseudomonas aeruginosa* are the primary bacteria responsible for otitis externa, while upper respiratory tract flora, such as *Streptococcus pneumoniae*, *Haemophilus influenzae,* and *Moraxella catarrhalis,* are the primary causative agents of otitis media. Recently, *Corynebacterium otitidis* (formerly *Turicella otitidis*) has become a recognized pathogen in both of these conditions (24). Rarely, other *Corynebacterium* spp., such as *C. amycolatum*, *C. auris*, *C*. *propinquum*/*pseudodiphtheriticum* group, have been associated with ear infections (7, 25).

#### Intra-abdominal infections

The major intra-abdominal infection caused by *Corynebacterium* spp. is peritoneal dialysis-associated peritonitis (26, 27). This is likely due to biofilm formation on the dialysis catheter from these skin-dwelling organisms. *C. amycolatum, C. jeikeium, *and *C. striatum* are the most common causative agents, but cases involving *C. aurimucosum*/*minutissimum* group, *C. propinquum*/*pseudodiphtheriticum* group*, *and* C. ulcerans* have been reported (26, 27). These infections can be challenging to treat with antimicrobial therapy alone and often relapse; catheter removal for source control is frequently necessary (26).

Spontaneous bacterial peritonitis due to *Corynebacterium* spp. is rare but does occur (28). Several cases of infection of pancreatic pseudocysts by *Corynebacterium* species (*C. striatum* and *C. xerosis*) have been described (29, 30). Pancreatic and liver abscesses due to *Corynebacterium* species have also been reported (7, 31, 32).

### Musculoskeletal infections

#### Native bone and joint infections

Historically, *Corynebacterium* species have largely been considered non-pathogens in most orthopedic infections. This is no longer the case as there has been an increasing recognition of true infections in bones and joints due to these bacteria (33). *C. accolens, C. amycolatum, C. aurimucosum*/*minutissimum* group*, C. diphtheriae* complex*, C. jeikeium*, *C. macginleyi*, *C. propinquum*/*pseudodiphtheriticum* group, *C. simulans, C. striatum, C. tuberculostearicum* complex*, C. ulcerans*, *C. urealyticum, *and* C. xerosis* have been recovered from native bone and joint infections (7, 33–35). These infections often follow trauma and/or orthopedic surgery, but spontaneous cases have occurred as well (7, 33–35).

#### Periprosthetic joint and orthopedic hardware-associated infections

Similar to many other cutaneous flora, *Corynebacterium* species have a large role in periprosthetic joint and orthopedic hardware-associated infections (7, 36). *C. striatum* is the predominant coryneform bacterium isolated from these infections (7, 36)*. C. amycolatum*, *C. aurimucosum*/*minutissimum* group*, C. bovis, C. jeikeium, *and* C. massiliense* have also been associated with prosthetic joint infections (7, 36).

### Respiratory tract infections

Diphtheria toxin-producing *Corynebacterium* spp. are well-known pulmonary pathogens known to cause both upper and lower respiratory tract infections (4). Historically, the most associated infection with these bacteria is respiratory diphtheria, resulting in pseudomembranous pharyngitis (4). Systemic effects due to the toxin are a known complication of this disorder, which can result in death (4). Fortunately, vaccination and the availability of antitoxin have dramatically lowered the morbidity and mortality associated with this pathogen (4). Pneumonia due to *C. diptheriae* complex, *C. pseudotuberculosis, *and* C. ulcerans* can occur from both toxigenic and non-toxigenic strains (4, 7).

Infections of the respiratory tract due to non-DT-producing *Corynebacterium* spp. have become a recognized issue in recent years (37). It is now known that these bacteria can cause pulmonary infections in certain clinical scenarios and patient populations (37). This is most seen in patients with chronic lung disease, immunosuppressed hosts, and/or critically ill patients requiring mechanical ventilation (37). The *C. propinquum*/*pseudodiphtheriticum* group is a common colonizer of the human respiratory tract but is also the most common cause of pneumonia due to *Corynebacterium* species (37). *C. striatum* can be associated with pneumonia (37). *C. accolens*, *C. afermentans*, *C. jeikeium*, *C. macginleyi, C. mucifaciens*, *C. simulans, C. sputi, C. urealyticum*, and *C. xerosis* have been known to cause pneumonia rarely (7, 37, 38)*. C. argentoratense* and *C. imitans* have been attributed to upper respiratory tract infection (7). Given that *Corynebacterium* spp. are common colonizers of nasal sinuses, determining if they are pathogens in cases of rhinosinusitis can be challenging. *C. accolens, C. propinquum*/*pseudodiphtheriticum* group*, *and* C. tuberculostearicum* complex are possible causes of rhinosinusitis when a shift in bacterial flora occurs (39).

### Urinary tract infections

Contamination of urine samples submitted for culture with endogenous mucocutaneous flora, such as *Corynebacterium* spp., is common. Due to this, *Corynebacterium* species are often dismissed in urine cultures. However, certain species can be urinary pathogens and should not be so easily disregarded. This is especially true for urease-producing members of this genus.

Encrusted cystitis and encrusted pyelonephritis occur when the urease activity of these bacteria leads to urinary alkalinization and precipitation of inorganic salts (40). These inorganic salts adhere to the urothelial epithelium, forming encrustations in the bladder and/or kidney, leading to abdominal pain, dysuria, and necrosis, and can lead to renal failure in severe cases (40). Risk factors include immunosuppression, chronic illness, previous antibiotic use, and history of previous genitourinary disorder or instrumentation (40). *C. urealyticum* is the most common pathogen associated with this condition (40). It is lipophilic and can be slow-growing in culture, making microbiological recovery challenging if not screened for in urine cultures (7). This can result in a delay in diagnosis and initiation of appropriate antimicrobial therapy (40). Obstructive uropathy resulting in hyperammonemia has been seen with *C. urealyticum* as well as with other urease-producing *Corynebacterium* species such as *Corynebacterium propinquum*/*pseudodiphtheriticum* group and *Corynebacterium reigelii *(41, 42)*. C. glucuronolyticum *and* C. reigelii* have been frequently implicated in urinary tract infections (6, 7, 40, 41). Urinary tract infections have also been described due to *C. amycolatum, C. aurimucosum*/*minutissimum* group*, C. coyleae, C. jeikeium, C. macginleyi, C. tuberculostearicum* complex, and other urease-producing species (6, 7).

### Lymphadenitis

Generally caused by *C. pseudotuberculosis*, caseous lymphadenitis is a major disease in sheep, goats, and other ruminants (3, 7). Occasionally, humans acquire the disease from an animal source (3, 7). Lymphadenitis has also been described in infections caused by other *Corynebacterium* species (7).

## LABORATORY IDENTIFICATION & SUSCEPTIBILITY TESTING

The ability of the laboratory to accurately identify bacteria belonging to the genus *Corynebacterium* to the species level has grown over the past several decades (7, 9).

Throughout most of the 20th century, identification of *Corynebacterium* spp. to the species level required the use of a variety of biochemical and phenotypic methods (7, 9). This testing can be challenging and time-consuming, especially for the fastidious members of this genus (7). Performing this testing was impractical for most clinical laboratories outside of large reference centers (7). Due to this, species-level identification was not generally performed, and laboratories often reported them with a morphological description such as “coryneform Gram-positive rods” or “diphtheroids” when there was little clinical concern for diphtheria (9). In cases where there was a concern for diphtheria, the bacterial isolate could be cultivated on a differentiation media, such as Tinsdale or Loeffler medium (9). On Tinsdale medium, *C. diphtheriae* complex and *C. ulcerans* will appear as grayish-black colonies surrounded by a brown/black halo, this colony appearance can rarely be seen in other *Corynebacterium* species and other Gram-positive bacteria (9). *Corynebacterium* species appear as cream-colored colonies with slightly raised centers when grown on Loeffler medium. Metachromatic granules can be visualized when colonies grown on Loeffler medium are viewed microscopically after methylene blue staining; however, this is a non-specific finding for *C. diphtheriae* complex, other *Corynebacterium* spp., and Gram-positive bacteria may also produce these granules (25). Species-level identification cannot be obtained from the use of these differentiative media.

As it became clinically apparent that *Corynebacterium* spp. other than *C. diphtheriae* complex (diphtheroids) can cause serious infections over the last several decades, the need for clinical microbiology laboratories to perform species-level identification in certain situations has become necessary (7, 9). In the 1990s, rapid biochemical test panels such as the API Coryne (Biomérieux Inc., Durham, NC), RapID CB plus system (Remel Inc., Lenexa, KS), and the ANC identification card for the Vitek 2 automated system (Biomérieux, Marcy I’Étoile FR) became available, allowing most clinical labs the ability to identify isolates to the species complex level (7). Although useful, these biochemical-based tests can result in misidentification and are not as reliable as molecular and proteomic-based methods (14, 15).

In the last decade or so, new technological advances have allowed the rapid, inexpensive, and accurate identification of these organisms using molecular and proteomic techniques in many clinical microbiology laboratories. Matrix-assisted laser desorption/ionization time-of-flight mass spectrometry (MALDI-TOF MS) is now widely used in clinical microbiology and allows for the identification of most pathogenic coryneform gram-positive rods using an FDA-cleared method. Organism identification claims are submitted to the FDA for clearance by the MALDI-TOF MS device manufacturers. Claimed organisms have been determined by the FDA to result in reliable identification by the device to the submitted taxonomic level (genus, species, or species group/complex). This is largely based on which spectral reference library is used. Thus, claimed organisms have been FDA-cleared for identification utilizing the MALDI-TOF MS with the corresponding spectral reference library. The most current FDA-cleared reference libraries are the Bruker MALDI Biotyper CA reference library claim 6 (2020) and the bioMérieux VITEK MS knowledge base v3.3 (2024). Table 1 compares the claimed *Corynebacterium* species available for the two commercially available MALDI-TOF MS platforms on their most recently available FDA-cleared reference libraries. Microbiology laboratories may also have validated research libraries to expand their diagnostic capabilities; however, this would be considered a laboratory-developed test requiring extensive validation of the reference libraries’ clinical performance.

**TABLE 1:** Comparison of MALDI TOF MS FDA 510(k) Cleared *Corynebacterium* species identification

Bruker MALDI Biotyper CA Reference Library Claim 6 (2020)	Biomérieux Vitek MS Knowledge Base v3.3 (2024)
*Corynebacterium accolens*	*Corynebacterium accolens*
*Corynebacterium afermentans* group	*Corynebacterium afermentans* ssp *afermentans*
*Corynebacterium amycolatum*	*Corynebacterium afermentans* ssp *lipophilum*
*Corynebacterium argentoratense*	*Corynebacterium amycolatum*
*Corynebacterium aurimucosum* group	*Corynebacterium argentoratense*
*Corynebacterium bovis*	*Corynebacterium aurimucosum*
*Corynebacterium confusum*	*Corynebacterium auris*
*Corynebacterium coyleae*	*Corynebacterium auriscanis*
*Corynebacterium diphtheriae*	*Corynebacterium bovis*
*Corynebacterium durum*	*Corynebacterium confusum*
*Corynebacterium freneyi*	*Corynebacterium coyleae*
*Corynebacterium glucuronolyticum*	*Corynebacterium cystitidis*
*Corynebacterium glutamicum*	*Corynebacterium diphtheriae*
*Corynebacterium imitans*	*Corynebacterium durum*
*Corynebacterium jeikeium*	*Corynebacterium falsenii*
*Corynebacterium kroppenstedtii*	*Corynebacterium freneyi*
*Corynebacterium macginleyi*	*Corynebacterium glucuronolyticum*
*Corynebacterium minutissimum*	*Corynebacterium glutamicum*
*Corynebacterium mucifaciens/ureicelerivorans group*	*Corynebacterium glyciniphilum*
*Corynebacterium propinquum*	*Corynebacterium imitans*
*Corynebacterium pseudodiphtheriticum*	*Corynebacterium jeikeium*
*Corynebacterium pseudotuberculosis*	*Corynebacterium macginleyi*
*Corynebacterium resistens*	*Corynebacterium mastitidis*
*Corynebacterium riegelii*	*Corynebacterium matruchotii*
*Corynebacterium striatum group*	*Corynebacterium mucifaciens*
*Corynebacterium tuberculostearicum*	*Corynebacterium otitidis*
*Corynebacterium ulcerans*	*Corynebacterium pilosum*
*Corynebacterium urealyticum*	*Corynebacterium propinquum*
*Corynebacterium xerosis*	*Corynebacterium pseudotuberculosis*
	*Corynebacterium renale*
	*Corynebacterium riegelii*
	*Corynebacterium simulans*
	*Corynebacterium stationis*
	*Corynebacterium striatum*
	*Corynebacterium sundsvallense*
	*Corynebacterium timonense*
	*Corynebacterium tuberculostearicum*
	*Corynebacterium ulcerans*
	*Corynebacterium variabile*
	*Corynebacterium xerosis*

Although the most recent FDA-cleared reference libraries can reliably identify most of the commonly encountered *Corynebacterium* species in clinical settings, some rarely encountered species cannot be. For comparison, the Vitek MS (Knowledge Base v3.3) currently has 40 claimed identifications while the MALDI Biotyper (CA Reference Library Claim 6) has 29 (Table 1). The clinical performance of these most recent reference libraries for the identification of *Corynebacterium* species has not been extensively evaluated. Previous studies on the clinical performance of MALDI-TOF MS in the identification of *Corynebacterium* spp. have shown challenges in differentiating some closely related species, resulting in species misidentification (14, 15). These studies showed issues with differentiating certain species, such as *C. aurimucosum* from *C. minutissimum* and *C. minutissimum* from *C. singular* (14, 15). The method of preparing the isolate for MALDI-TOF MS analysis impacts the identification ability of the instrument (6). The direct colony transfer method is highly effective; however, using the on-target extraction method, with the addition of formic acid, does improve the quality of the spectrum obtained (15). The tube extraction method can be used if the above techniques fail, but it is a time-consuming, more technically complex process (14). The previous studies on the performance of MALDI-TOF MS utilized older reference libraries with limited spectra in their reference libraries (14, 15). New clinical studies evaluating the performance of the recent FDA-cleared reference libraries are needed. In cases where the bacterial isolates' colony morphology and growth characteristics do not match the identification generated by MALDI-TOF MS, sequencing may be considered for definitive species-level identification. This is especially true for species known for misidentification by MALDI-TOF MS (14, 15).

In larger clinical laboratories with advanced molecular capabilities, sequencing of the 16S rRNA and *rpoB* genes allows for accurate and precise identification results to species/species complex level in many cases (15). However, sometimes, these sequencing gene targets are not able to accurately identify *Corynebacterium* to the species level. Whole-genome sequencing (WGS) is likely required to differentiate some closely related species within the genus (43). This results in the reliance on WGS for species-level molecular diagnosis of these difficult-to-identify *Corynebacterium* spp. such as *C. aurimucosum*/*minutissimum* and *C. propinquum*/*pseudodiphtheriticum*. Due to this, some of the organisms previously identified by alternative methodologies have likely been misidentified as a closely related species. Alternative gene targets and unique genetic loci that can be elucidated by WGS have been characterized and may aid in species identification for some *Corynebacterium* spp.: (i) In pigmented strains of the *C. aurimucosum*/*minutissimum* group (*C. nigricans*), the production of the black pigment has been associated with genes identified in the pET44827 plasmid (44). These gene functions are hypothesized to protect the organism in the elevated hydrogen peroxide concentrations observed in the vagina (44). (ii) Isolates of *C. propinquum* are found to have a gene copy of the virulence factor isocitrate lyase, as well as the transporter gene, arsenical-resistance protein ACR_3_ (45). In addition, the presence of *erm(X*), *cmx*, and *Sul1* genes has been identified in isolates of *C. propinquum* showing resistance to macrolides-lincosamides-streptogramins, chloramphenicol, and sulfonamides. (iii) The presence of phospholipase D and neuraminidase genes has been characterized in virulent strains of *C. pseudotuberculosis* and *C. ulcerans* (46). (iv) Compared to nontoxigenic strains, an intact, toxin-producing strain of *C. diptheriae* has been characterized to have an additional 11 pathogenicity islands and 37 unique genetic regions (47). (v) Facilitation of urine alkalization and struvite stone formation of *C. urealyticum* has been associated with a specialized urease gene locus (48).

**Fig 1:**
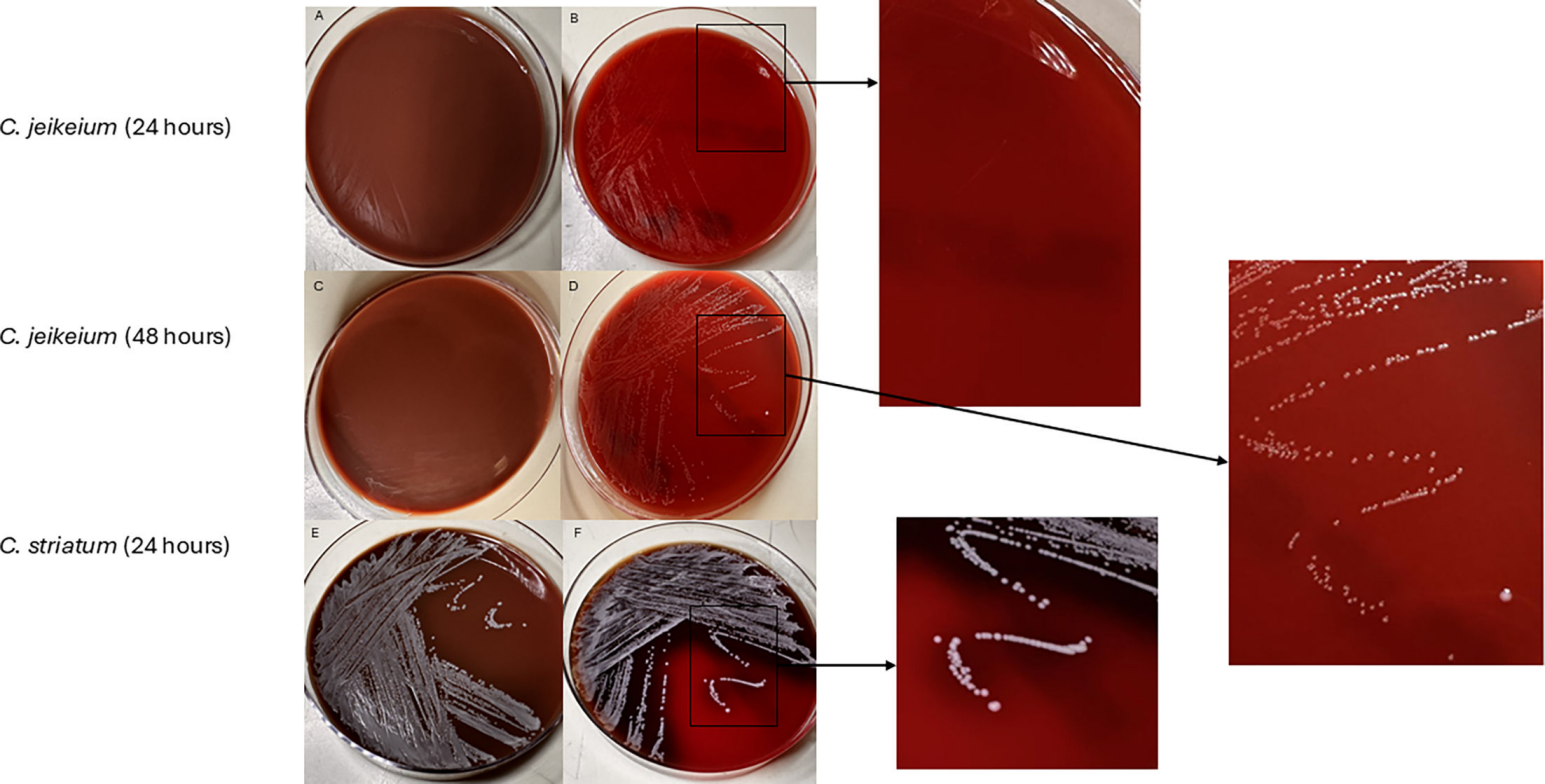
Images of secondary culture of *C. jeikeium* (lipophilic) and *C. striatum* (nonlipophilic) inoculated onto chocolate agar (left column) and blood agar (right column) and incubated aerobically at 35°C for 24–48 hours. Lipophilic *Corynebacterium* spp. grow poorly on media that lack lipid supplementation, such as chocolate agar (A and B), but grow better on blood agar (C and D), where the red cell membranes can provide the lipids needed for growth. They often need extended incubation and will often have a hazy growth appearance (A) in the first 24 hours of incubation before forming discrete but tiny colonies (B) at 48 hours. Nonlipophilic *Corynebacterium* spp. grow equally well on chocolate and blood agar (E and F).

A challenge laboratories face in the recovery of pathogenic *Corynebacterium* spp. is the lipophilic nature of some of these bacteria. (Table 2) The lipophilic members of this genus grow poorly on most routine media used in clinical microbiology laboratories due to the lack of ideal lipid concentrations. They rarely grow efficiently on chocolate agar and will have hazy growth or tiny colonies on blood agar (Fig. 1). This may lead to ineffective recovery of these pathogens from routine bacterial culture media setup schemes. Colonies suspected to be lipophilic *Corynebacterium* spp. (pinpoint/tiny colonies) can be subcultured to brain-heart infusion agar (BHI) or blood agar with a sterile olive oil overlay applied for lipid supplementation to increase their propagation in culture. These bacteria, which are highly related to *Mycobacterium* spp., can occasionally be recovered in acid-fast bacilli (AFB) culture due to lipid concentrations in the broth and agar media preparation (e.g., Middlebrook) used to isolate these similarly lipophilic organisms.

**TABLE 2:** Characteristics of pathogenic *Corynebacterium* species^*a*^

	Corynebacterium species	Pathogenicity	Frequency	Lipophilic	Zoonotic
DT-producing Corynebacterium species	*C. belfantii*	Diphtheria	Uncommon	No	No
	*C. diphtheriae*	Abscess, BJI, BSI, diphtheria, endocarditis, ocular infections, URTI	Common	No	No
	*C. pseudotuberculosis*	Caseous lymphadenitis, diphtheria	Common	No	Yes
	*C. riouxii*	Diphtheria	Uncommon	No	Yes
	*C. silvaticum*	Diphtheria	Uncommon	No	No
	*C. ulcerans*	Abscess, BJI, BSI, diphtheria, ocular infections, PD-associated peritonitis, URTI	Common	No	Yes

Non-DT-producing Corynebacterium species	*C. accolens*	Abscess, BJI, BSI, ocular infections, pulmonary infections	Common	Yes	No
	*C. afermentans* subsp. afermentans	Abscess, BSI,	Common	No	No
	*C. afermentans* subsp. lipophilum	Abscess, BSI, IAI,	Common	Yes	No
	*C. amycolatum*	Abscess, BJI, BSI, cutaneous infections, GLM, ear infections, endocarditis, ocular infections, PD-associated peritonitis, PJI, skin/soft tissue abscess, UTI	Common	No	Yes
	*C. argentoratense*	BSI, URTI	Uncommon	No	No
	*C. aurimucosum*/minutissimum group	BJI, BSI, cutaneous infections, CSF shunt infection (49), endocarditis, erythrasma, meningitis, PD-associated peritonitis, PJI, UTI	Common	No	No
	*C. auris*	Ear infections	Uncommon	No	No
	*C. bovis*	Brain abscess (50), CSF shunt infection (50), endocarditis (50), meningitis, ocular infections, PJI	Common	Yes	Yes
	*C. canis*	Dog bite-related wound	Uncommon	No	Yes
	*C. coyleae*	Abscess, BSI, endocarditis, IAI, UTI	Uncommon	No	No
	*C. falsenii*	BSI	Uncommon	No	Yes
	*C. flavescens*	Trichobacteriosis	Uncommon		
	*C. freiburgense*	Dog bite-related wound	Uncommon	No	Yes
	*C. freneyi*	BSI, endocarditis (51)	Uncommon	No	No
	*C. glucuronolyticum*	BSI, ocular infections (52), UTI	Uncommon	No	Yes
	*C. haemomassiliense* group	BSI (53)	Uncommon	No	No
	*C. imitans*	Pharyngitis, BSI, URTI	Uncommon	No	No
	*C. jeikeium*	BJI, BSI, CSF shunt infection, cutaneous infections, ear infections (54), endocarditis, GLM (55), IAI, meningitis, ocular infections, PD-associated peritonitis, PJI, pulmonary infections, UTI	Common	Yes	Yes
	*C. kroppenstedtii*	Breast abscess, BSI, endocarditis (56), GLM	Common	Yes	No
	*C. kutscheri*	Rat bite-related wound	Uncommon	No	Yes
	*C. lipophilum*	Mastitis (57)	Uncommon	Yes	No
	*C. macginleyi*	BJI, BSI, cutaneous infections, endocarditis, ocular infections, pulmonary infections, UTI	Common	Yes	No
	*C. massiliense*	PJI	Uncommon	No	No
	*C. mastitidis*	Ocular infection	Uncommon	Yes	Yes
	*C. mucifaciens*	BSI, ear infections (58), pulmonary infections	Uncommon	No	No
	*C. mycetoides*	Cutaneous ulcers	Uncommon	No	No
	*C. nuruki*	BSI (59)	Uncommon	Yes	No
	*C. otitidis*	BSI (60), ear infections, ocular infections (61)	Common	No	No
	*C. parakroppenstedtii*	GLM	Uncommon	Yes	No
	*C. phoceense*	UTI (62)	Uncommon	No	No
	*C. propinquum*/pseudodiphtheriticum group	BJI, brain abscess (63), BSI, cutaneous infections, endocarditis, lymphadenitis, ocular infections, PD-associated peritonitis, pulmonary infections, trichobacteriosis, UTI	Common	No	No
	*C. pseudogenitalium*	UTI (64)	Uncommon	Yes	
	*C. pseudokroppenstedtii*	GLM	Uncommon	Yes	No
	*C. pyruviciproducens*	Abscess	Uncommon	No	No
	*C. resistans*	Brain abscess, BSI, cutaneous infections	Uncommon	Yes	No
	*C. riegelii*	UTI	Uncommon	No	No
	*C. simulans*	BJI, BSI, endocarditis, pulmonary infections	Uncommon		
	*C. sputi*	Pulmonary infection	Uncommon	Yes	No
	*C. striatum*	Abscess, BJI, BSI, CSF shunt infection, cutaneous infections, ear infections (65), endocarditis, IAI, meningitis (66), ocular infections, PD-associated peritonitis, PJI, pulmonary infections, UTI	Common	No	No
	*C. timonense*	BSI, endocarditis	Uncommon	No	No
	*C. tuberculostearicum* complex	BJI, GLM, lymphadenitis (67), UTI	Uncommon	Yes	No
	*C. tuscaniae*	BSI, endocarditis	Uncommon	No	No
	*C. urealyticum*	BSI, endocarditis, pulmonary infections, UTI	Common	Yes	Yes
	*C. ureicelerivorans*	BSI	Uncommon	Yes	No
	*C. xerosis*	Abscess, BJI, brain abscess, BSI, CSF shunt infection, ear infections (54), endocarditis, IAI, meningitis, ocular infections, pericarditis, pulmonary infections, SBP	Common	No	Yes

^*a*^ Abbreviations: BJI, bone/joint infection; BSI, bloodstream infection; CSF, cerebrospinal fluid; GLM, granulomatous lobular mastitis; IAI, intra-abdominal infection; MDR, multidrug resistant; PD, peritoneal dialysis; PJI, prosthetic joint infection; URTI, upper respiratory tract infection; UTI, urinary tract infection.

### Antimicrobial susceptibility testing

Given the fastidious nature of many *Corynebacterium* species, performing susceptibility testing may be challenging for many clinical laboratories. For laboratories with technical expertise and the capability to perform this testing, it may be beneficial if the laboratory services a large volume of immunosuppressed patients and those with implanted prosthetic joints (68, 69). Susceptibility testing for *Corynebacterium* species can be performed utilizing the Clinical and Laboratory Standards Institute (CLSI) M45-ED3 (2016) manual for guidance (68, 69). Microbroth dilution with cation-adjusted Mueller-Hinton broth supplemented with lysed horse blood (CAMHB-LHB) (2.5%–5% vol/vol) can be used to test several antimicrobial agents, including vancomycin, linezolid, doxycycline, penicillin, ceftriaxone, meropenem, and ciprofloxacin (68, 69). Daptomycin can also be tested utilizing this method by supplementing the CAMHB-LMB with 50 µg/mL (68, 69). Some laboratories have opted to utilize E-test (bioMérieux, Durham, NC) as an alternative to the CLSI-recommended microbroth dilution technique (69). The test medium needs to be incubated at 35°C in ambient air for 20–24 hours, with beta-lactam antibiotics requiring 24–48 hours of incubation (68, 69).

There appears to be significant variability in the susceptibility patterns of non-DT-producing *Corynebacterium* spp. further necessitating the need for antimicrobial susceptibility testing (AST) when targeted therapy is required (68, 69). *Corynebacterium* species can be considered universally susceptible to vancomycin and linezolid agents (6, 68, 69). AST is not generally needed if these two agents are used for antimicrobial therapy. Tigecycline also appears to have high susceptibility in *Corynebacterium* isolates that underwent AST (68). Unfortunately for these organisms, susceptibility to many oral options has decreased over the past several years (69). Penicillin, clindamycin, and erythromycin have all seen a substantial decrease in the number of susceptible isolates that have been tested (6, 69). *C. striatum,* in particular, has shown low susceptibility rates to most oral options outside of linezolid (100%) and occasionally doxycycline (24%) (69). Doxycycline, trimethoprim-sulfamethoxazole, and clindamycin may be considered as alternative oral options for non-*C*. *striatum* species; however, AST should be considered if used as monotherapy (6, 69). Elevated minimum inhibitory concentration (MIC) values toward daptomycin have also been observed in *C. jeikeium* (68, 69).

Antimicrobial resistance mechanisms tend to be either unknown or varied within the *Corynebacterium* genus (69). The exact mechanism of daptomycin resistance in *Corynebacterium* is not well understood (68). Most *Corynebacterium* species carry a beta-lactamase gene (*bla*), while *C. striatum* has also been seen to harbor genes encoding an ampC enzyme (6). Gentamicin resistance in *C. striatum* is associated with the *aac(3)-XI* gene, which encodes an aminoglycoside 3-N-acetyltransferase (6, 68). Erythromycin and clindamycin resistance is largely mediated through the *ermX* gene (6, 68). Mutations to the gyrase A gene (*gyrA*) are seen to frequently confer resistance to ciprofloxacin (6). The *tetA* and *tetB* genes have been commonly found in non-DT-producing *Corynebacterium* species, resulting in varying levels of resistance toward tetracyclines (6).

## DISCUSSION

Historically, *Corynebacterium* species and related genera, excluding *C. diphtheriae*, have been considered non-pathogenic flora of mucocutaneous surfaces. Given this belief and the challenges in identifying these bacteria to genus/species level utilizing biochemical methods, clinical laboratories often reported them as “coryneform gram-positive rods (GPRs)” or “diphtheroids,” if not just lumped them in with other skin flora if present (9). The routine use of newer technologies in clinical microbiology laboratories, such as MALDI-TOF MS and DNA sequencing, has allowed the rapid and accurate identification of these organisms in a cost-effective manner (7).

Although many clinical microbiology laboratories can now fully identify coryneform GPR to the species level, determining when they should do this is the current challenge. The vast majority of coryneform GPR encountered in clinical microbiology labs are either contaminants, colonizers, or part of polymicrobial infections where general empirical therapy is most appropriate. Our criteria for identifying these bacteria in clinical cultures should be based on our knowledge of which patient populations and clinical situations are at risk for infections from *Corynebacterium* species. In addition, some species can be extensively antimicrobial resistant to most agents other than vancomycin, daptomycin, and linezolid (68, 69) (Table 2). Complicating this is the fact that *Corynebacterium* spp. can develop high-level daptomycin resistance even from short-term exposure to the drug (68, 69). This results in limited treatment options for these multidrug-resistant species.

Most *Corynebacterium* spp. isolated from blood culture are contaminants due to ineffective skin preparation prior to venipuncture; however, an increasing number of cases of bloodstream infections, including infective endocarditis and central line-associated bloodstream infections, are being reported due to these commensal bacteria. For facilities with access to MALDI-TOF MS, performing species-level identification should be considered for blood isolates, as certain species, such as *C. striatum* and *C. jeikeium*, are associated with high morbidity and mortality with bloodstream infections (5). Due to the multidrug-resistant nature of both of these species, AST should be considered if an antimicrobial agent other than vancomycin is to be used for treatment. For facilities without access to MALDI-TOF MS, species-level identification should be considered when coryneform GPRs are isolated from multiple blood culture sets within a 24 hour period, single-set blood cultures, or in patients with high clinical concern for endovascular or hematological infections (8, 10). Species-level identification in these scenarios should be performed for coryneform GPR that produce tiny, pinpoint colonies (*C. jeikeium*) and those that produce large, white-grayish colonies that are non-hemolytic with an entire edge (*C. striatum*) (9). Due to the unreliability of phenotypic testing, sending these isolates to reference laboratories where a reliable species-level identification can occur should be considered (14, 15).

Like blood cultures, CSF cultures are often contaminated by these bacteria, especially when obtained by lumbar puncture. Cases of community-acquired meningitis are exceedingly rare; most cases involve patients with a history of neurosurgery and/or CSF shunt placement. In cases where the isolate is likely a contaminant, species-level identification and susceptibility testing are often unnecessary. For patients with this history of CNS instrumentation, including indwelling CSF shunt placement, coryneform GPR isolated from CSF should be identified to species level, as their propensity toward biofilm formations makes them major CNS pathogens in this patient population (6). Certain species are more virulent and multidrug-resistant, which may alter the treatment regimen and duration of therapy. AST should be strongly considered in cases where vancomycin or linezolid are not used as one of the therapeutic agents.

Reporting *Corynebacterium* spp. from wound cultures can be challenging. Most of the time, they represent contamination, superficial colonization of the wound, or a component of a mixed infection. The laboratory should be notified if the patient is immunosuppressed, as these patients may benefit from a more detailed culture workup strategy. Many electronic health record ordering systems allow for ordering questions where this information may be provided for laboratory review. Certain *Corynebacterium* species, such as *C. jeikeium* and *C. striatum*, can cause significant cutaneous infections in immunocompromised hosts that can subsequently lead to invasive infections (10). In these patients, the threshold to fully identify these bacteria should be much lower than in immunocompetent individuals. In patients with chronic, non-healing cutaneous ulcers, both *C. diphtheriae* complex and *C. ulcerans* may be investigated, especially in wounds where patients do not have classic risk factors (e.g., diabetes, pressure injury, vasculopathy) for chronic non-healing skin ulcers.

In deep tissue/bone cultures and sterile fluids, the full identification threshold should again be lower. It is important that definitive identification be performed in invasively and sterilely collected specimens when significant amounts of the organism are present in culture or when it is clearly the predominant organism. Correlation with direct specimen Gram stain can be helpful as it provides correlation with *in vivo* organism load in ambiguous cases. If the body site where the sample was collected communicates with any medical hardware or devices, any amount should be reported to species levels due to the biofilm formation capabilities of most *Corynebacterium* species (6). In breast tissue/aspirate samples, *C. kroppenstedtii* and other lipophiles that can be responsible for granulomatous lobular mastitis and breast abscesses should be screened for. Lipophilic coryneform GPR will appear as hazy growth or tiny colonies growing on blood agar plates that may take several days (2–3 days) to become discernible.

If targeted antimicrobial therapy is indicated for wounds and sterile body tissue/fluid samples, vancomycin and linezolid are reliable options for empirical coverage of *Corynebacterium* species. If an alternative agent is utilized, AST can be considered in cases where treatment failure occurs or if prolonged therapy is anticipated. Laboratories that perform in-house susceptibility testing on GPR may consider performing susceptibility testing on clinically significant isolates. Due to the costs associated with reference laboratory testing and the high volume of isolates, relying on clinicians’ requests for AST may be the best practice for most facilities that do not perform this testing.

For ocular sites, *C. macginleyi* should be actively screened for by clinical microbiologists. This can be accomplished by screening for lipophilic *Corynebacterium* species (tiny colonies on blood agar) in eye cultures. Similarly, in ear cultures, the otic pathogen *C. turicella* should be evaluated when a coryneform GPR is encountered in a significant amount or if it predominates over other microorganisms in culture. These infections are often treated with topical antimicrobial agents. Due to the high concentration of antibiotics in these preparations, clinical resistance is rarely encountered. Unless systemic therapy is needed, AST is generally not needed.

The presence of urease-producing *Corynebacterium* species, such as *C. urealyticum* and *C. reigelii*, should be ruled out when a significant level of a coryneform GPR (≥10,000 cfu/mL) is encountered in a urine sample or any amount from procedurally obtained urines. These pathogens are often disregarded in urine cultures as contaminants, which could lead to a delay in the diagnosis of encrusted cystitis. If rapid identification through MALDI-TOF MS is not available, rapid urease testing can be considered. For cases with a short treatment duration, such as acute cystitis and pyelonephritis, AST may not be required. Empirical therapy with vancomycin or linezolid is most appropriate for multidrug-resistant species such as *C. urealyticum*. For species not associated with major antimicrobial resistance, empirical therapy with beta-lactams, doxycycline, or fluoroquinolones may be considered. For cases that require prolonged treatment, AST is recommended to guide effective therapy, such as cases of encrusted cystitis.

These bacteria are also an emerging respiratory pathogen in critically ill patients. The organisms can colonize the lungs and trachea of these patients, making interpretation of clinical significance challenging. *C. propinquum*/*pseudodiphtheriticum* group and *C. striatum* are two species that may warrant reporting at high levels in respiratory cultures. At low levels in mixed cultures, reporting as normal oropharyngeal flora is likely the best practice. Given the potential severity of these infections in these patient populations, vancomycin and linezolid should be utilized if treatment is indicated.

Over the past several decades, the clinical importance of non-diphtheriae *Corynebacterium* species has become readily apparent. Optimization of clinical laboratory reporting is needed for *Corynebacterium* species, especially in facilities that serve immunosuppressed patient populations and/or high-volume prosthetic joint orthopedic services. It is also important that microbiologists understand the body site pathogenic niche of many of these bacteria to better screen for these pathogens. With the expanded use of MALDI-TOF MS, more pathogenic *Corynebacterium* species and other coryneform GPRs are likely to be described in the future.
